# Disrupting the Acyl Carrier Protein/SpoT Interaction *In Vivo*: Identification of ACP Residues Involved in the Interaction and Consequence on Growth

**DOI:** 10.1371/journal.pone.0036111

**Published:** 2012-04-30

**Authors:** Sandra Angelini, Laetitia My, Emmanuelle Bouveret

**Affiliations:** Laboratory of Macromolecular System Engineering (LISM), Centre National de la Recherche Scientifique (CNRS), Aix-Marseille University, Marseille, France; University of Delhi, India

## Abstract

In bacteria, Acyl Carrier Protein (ACP) is the central cofactor for fatty acid biosynthesis. It carries the acyl chain in elongation and must therefore interact successively with all the enzymes of this pathway. Yet, ACP also interacts with proteins of diverse unrelated function. Among them, the interaction with SpoT has been proposed to be involved in regulating ppGpp levels in the cell in response to fatty acid synthesis inhibition. In order to better understand this mechanism, we screened for ACP mutants unable to interact with SpoT *in vivo* by bacterial two-hybrid, but still functional for fatty acid synthesis. The position of the selected mutations indicated that the helix II of ACP is responsible for the interaction with SpoT. This suggested a mechanism of recognition similar to one used for the enzymes of fatty acid synthesis. Consistently, the interactions tested by bacterial two-hybrid of ACP with fatty acid synthesis enzymes were also affected by the mutations that prevented the interaction with SpoT. Yet, interestingly, the corresponding mutant strains were viable, and the phenotypes of one mutant suggested a defect in growth regulation.

## Introduction

In bacteria, Acyl Carrier Protein (ACP) is the free cofactor for type II fatty acid biosynthesis [1]. ACP is a small and acidic protein (8–10 kDa, pI≈4), which is also very abundant. It is modified post-translationnally on a conserved Serine residue (Serine 36 in *E. coli*) with a 4′-phosphopantetheine group (4′PP) by the holo-ACP synthase AcpS. Intermediates of fatty acid synthesis are constantly bound by a thioester link to the sulfhydryl group brought by the 4′PP group on ACP. As such, ACP interacts successively with and navigates between the numerous enzymes involved in fatty acid synthesis in order to deliver the acyl chain in elongation [2].

All the enzymatic steps of the fatty acid synthesis cycle have been extensively studied and are very well described [3]. In each case, an interaction between the enzyme and ACP is required, since acyl thioesters of ACP are the substrates of the reaction. In order to interact with so many distinct enzymes, to enable rapid association and dissociation, and to be able to shield or present the fatty acid chain to the enzymes, ACP structure must be highly flexible. Indeed, it has been shown to exist in different conformational states, with a flexible hydrophobic cavity [4], [5]. Furthermore, the specificity of recognition should not be too high, which is reflected by relatively weak Km of association [6], [7]. It has been shown that α-helix II of ACP plays a prominent role in this loose recognition of fatty acid synthesis enzymes [8], [9]. A synthetic peptide of 8 residues mimicking α-helix II and carrying an acyl-thioester is even sufficient for recognition by fatty acid synthesis enzymes [7]. This α-helix is rich in acidic residues, and is predicted to recognize patches of hydrophobic residues surrounded by basic residues on the surface of the enzymes [8].

Complexes and the corresponding 3D structures have been obtained only for AcpS and FabI in interaction with ACP [10], [11]. The structure of ACP in complex with the soluble STAS domain of the inner membrane protein YchM has also been described recently [12]. YchM is suggested to be a bicarbonate transporter and the interaction with ACP may be important to optimize fatty acid synthesis [12]. In addition, numerous partners of ACP were identified by Tandem Affinity Purification [13]–[15]. As expected, several enzymes involved in fatty acid or phospholipid biogenesis that are known to interact with ACP were present among them (such as the three keto-acyl synthases FabF, FabB, and FabH, but also FabD, FabG, AcpS, PlsB or Aas). However, several proteins that are not directly related to lipid metabolism (YbgC, IscS, MukB, and SpoT) were also identified in these ACP tandem affinity purifications. These interactions were further validated by co-purification experiments [13], [15], [16] but additional studies are still needed in order to understand their role in the cell.

We have previously characterized the interaction between ACP and SpoT [16]. SpoT is a bifunctional enzyme, that catalyses both hydrolysis and synthesis of (p)ppGpp, the effector of the stringent response in bacteria [17], [18]. (p)ppGpp is a global transcriptional regulator that binds RNA polymerase and impacts transcription of hundreds of genes. Upon nutritional starvation, (p)ppGpp level raises and as a consequence growth is arrested, mainly through inhibition of ribosome biogenesis. SpoT has been implicated in (p)ppGpp increase in response to a variety of starvation events, such as glucose, iron, phosphate, or fatty acid starvation [19]–[22]. Both activities of SpoT (ppGpp degradation and synthesis) are localized in the N-terminal half of the protein, and are controlled by allosteric transition shifting the protein from one activity to the opposite, which results in controlling (p)ppGpp level in the cell [23]. The C−terminal domain of SpoT is thought to be responsible for regulating this transition, however, the mechanism of this regulation is not understood. We had shown that ACP interacted with the C-terminal domain of SpoT, and that the ACP/SpoT interaction was involved in controlling the stringent response in response to fatty acid synthesis inhibition. These results suggested a regulation of SpoT catalytic activities by ACP, which may transduce the status of fatty acid metabolism in the cell to SpoT [16].

In this first study, we had looked for mutations in SpoT abolishing its interaction with ACP in order to study the role of the interaction [16]. However, a more direct proof of the regulation of SpoT by ACP would have been to find ACP mutants that did not interact with SpoT but that were still functional for fatty acid synthesis. We anticipated difficulties with this approach, due to the small size of ACP, its essentiality, and to the fact that it interacts with so many different enzymes for fatty acid synthesis. Yet, meanwhile, it was shown *in vivo* that ACP can tolerate a lot of point mutations [24]. Therefore, we decided to follow an approach of random mutagenesis on ACP, by looking for mutations abolishing specifically the interaction with SpoT. We aimed first at defining what is the region of interaction with SpoT on ACP, and then to characterize the physiological consequences of breaking the ACP/SpoT interaction.

## Results

### Screen for ACP mutants unable to interact with SpoT

**Figure 1:**
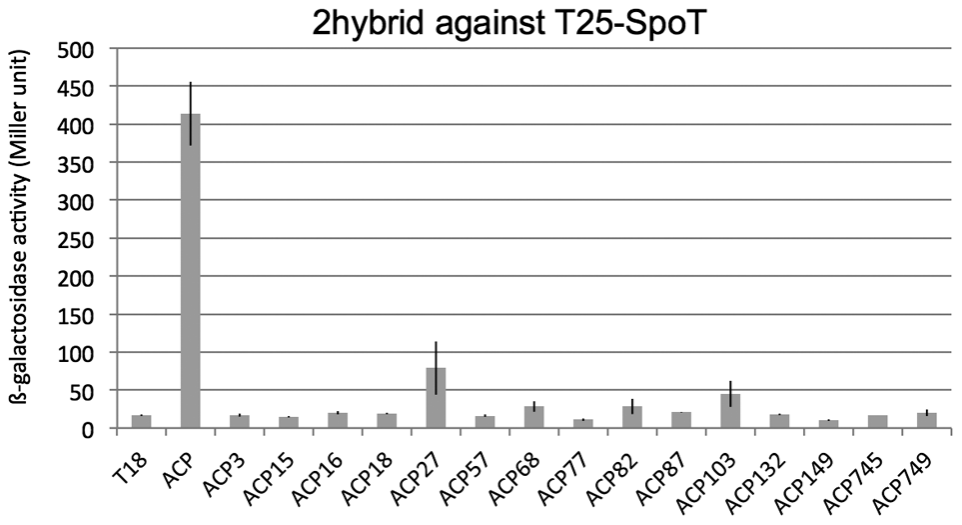
Bacterial two-hybrid assay between T25-SpoT and the series of T18-ACP mutants. BTH101 strain was transformed by pT25-SpoT (pEB595) and the series of pT18-ACP mutants isolated from the mutagenesis screen on MacConkey plates, and corresponding to full-length recombinant proteins. ß-galactosidase assay was performed on 3 independent clones for each pair, as described in the materials and methods.

In order to select mutants of ACP that could not interact with SpoT anymore, we used the bacterial two-hybrid technique that proved successful to characterize the ACP/SpoT interaction [16], [25], [26]. We performed random mutagenesis on the *acpP* coding sequence and then cloned it in the pT18link plasmid. The resulting library of pT18-ACP mutants was then screened by two-hybrid against pT25-SpoT and clones that had lost the interaction were selected (Materials and Methods). Expression of the recombinant T18-ACP mutant proteins was then verified by Western blotting using an antibody directed against the T18 domain. At this stage, more than half of the clones corresponded to truncated T18-ACP proteins, due to the deletion of one nucleotide caused by the mutagenesis process. We selected the clones giving full-length T18-ACP proteins (Figure S1). These clones were then sequenced (Table 1), and the loss of interaction with SpoT was again verified and quantified by ß-galactosidase assay (Figure 1 and data not shown).

**Table 1:** ACP mutants.

Clone number	Mutation(s)	Functionality
ACP3	D31Y-A45V	nd
**ACP15**, 23, 44, 45, 52, 56, 59, 63, 88, 135, 140	**M44I**	**+**
ACP16	G12C-A68D	nd
**ACP18**	**F50S**	+/−
ACP27	M44K	−
ACP50	L32P-T52S I69N	nd
ACP57	D51G-I54T	nd
ACP65	L15Q-K18R-L32P	nd
ACP67	E5K-S36P-F50S-I69N	nd
**ACP68**	**V43E**	**+**
ACP77	K9T-G16S-I62N	**−**
ACP82	A34P	**−**
**ACP87**	**T64A, ΔHQA, +12 residues**	**+**
ACP102	K8N-K9N-T23P-V65L	nd
ACP103	D35G-P55Q	nd
**ACP132**	**L42M**	**+**
ACP148	D31G-T39I-A45D	nd
ACP149	L32P	**−**
ACP745	A68D (from ACP 16)	**−**
**ACP749**	**I54T** (from ACP57)	**+/−**
ACP(S36T)	S36T	**−**

ACP mutants number 3 to 149 were obtained by random mutagenesis. Mutants ACP745 and ACP749 were obtained by site-directed mutagenesis. Mutant ACP(S36T) was described previously [15]. Functionality corresponds to the result of the complementation test by transduction of the Δ*acpP*::kana^R^ deletion in strain MG1655 containing the pKO3-*acpP* mutant construction (see text). + indicates growth on LB plate equivalent to the growth of the wild type; -indicates that no clone was obtained; +/− indicates that colonies were obtained, but growth was severely delayed; nd (not determined) indicates that the corresponding ACP mutants were not tested for functionality. The six mutants further characterized in the study are indicated in bold letters.

We obtained 28 clones for which the interaction was lost (Table 1). Among them, 11 clones contained the M44I mutation and 1 the M44K mutation. Besides the mutants on methionine 44, 5 additional clones contained a single mutation, and 11 clones contained 2, 3, or 4 mutations (Table 1). We discarded the mutants containing 3 or 4 mutations, but we wanted to test further the point mutations contained in the 4 clones containing two mutations (ACP3, ACP16, ACP103, ACP57). Therefore, we performed site directed mutagenesis on pT18-ACP plasmid in order to clone the 8 corresponding single mutations independently, and we checked again if the ACP mutants interacted with SpoT. This gave us 2 additional point mutants that did not interact with SpoT (ACP745 and ACP749, Table 1; Figure 1). Finally, the ACP87 clone containing the Thr64Ala mutation, a deletion of the three last residues of ACP, and an unrelated C-terminal 12 residues extension, was also selected due to its interesting properties (see below).

In total, at this step, we selected 10 single point mutants that did not interact with SpoT (ACP15, ACP18, ACP27, ACP68, ACP77, ACP82, ACP132, ACP149, ACP745, ACP749) plus ACP87 for further analysis (Table 1).

### Screen for ACP mutants that are still functional for fatty acid synthesis

The ACP mutants were screened for loss of interaction with SpoT. Yet, we wanted to test the effect of losing specifically this interaction, without losing essential interactions required for fatty acid biosynthesis. Indeed, *acpP* is essential, due to its role in fatty acid biosynthesis [27]. In order to test rapidly the functionality of the ACP mutants, we tried to complement a strain that contains an *acpP* ^ts^ allele [26], [27]. We first verified that the pT18-ACPwt construct was able to complement the MG1655*acpP* ^ts^ strain for growth at 42°C (data not shown, [26]), and then tested the 11 clones selected above. Only the ACP15(M44I) mutant was clearly able to complement MG1655*acpP* ^ts^ for growth at 42°C (data not shown). ACP68(V43E) and ACP132(L42M) mutants also complement to some extent as they allowed the formation of small colonies at 42°C, but there was no further growth after subcloning again at 42°C (data not shown). It has to be noted that the MG1655*acpP* ^ts^ strain is also deleted for the adjacent *fabF* gene. *fabF* is not essential [27], but it cannot be excluded that its deletion contributes to the defaults observed for the *acpP* mutants. Furthermore, in this assay, the functionality of the ACP mutants is tested at 42°C, while some mutants might prove to be thermosensitive yet functional at lower temperatures. Finally, the function of T18-ACP recombinant protein might already be non optimal as compared with wild type ACP.

In order to get rid of all the problems just exposed, we had to design a new genetic assay for getting a clear conclusion about the functionality of the ACP mutants. We decided to assay the possibility of P1 transduction of a Δ*acpP*::kana^R^ allele in strains transformed with plasmids expressing the ACP mutants (this is similar to an assay previously used for testing cyclic ACPs activity [28]). The *acpP* gene with its wild type promoter sequence was cloned in the low copy pKO3 vector [29]. We transformed the MG1655 strain by the pKO3-*acpP* plasmid (pEB1334), and we replaced the *acpP* ORF in this strain by a kanamycin resistance cassette, giving strain MG1655Δ*acpP*::kana^R^/pKO3-*acpP* (EB689). The wild type MG1655 strain was then transformed by the pKO3-*acpP* mutant plasmids. On these strains, the possibility to transduce the Δ*acpP*::kana^R^ allele from EB689 strain was finally tested.

We obtained clones for the ACP15(M44I), ACP68(V43E), ACP87(T64A+Ct), and ACP132(L42M) mutants, demonstrating that they were functional for fatty acid synthesis (Table 1). We also obtained clones for the ACP18(F50S) and ACP749(I54T) mutants, yet they were severely affected in growth. As a negative control, we verified that it was impossible to delete *acpP* in a strain transformed with pKO3-*acpP*(S36T) coding for an ACP(S36T) mutant that cannot be post-translationnally modified by 4′-PP and is therefore not functional.

**Figure 2:**
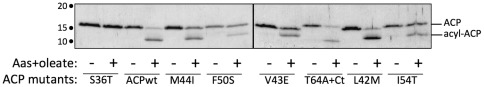
In vitro acylation assay of purified ACP mutants. The indicated purified ACP mutants were assayed for acylation with oleate using acyl-transferase Aas enzyme (see Material and Methods). ACP species were analyzed on a 15% SDS-PAGE stained with Coomassie Blue. Molecular weight markers in kDa are indicated on the left-hand of the gel.

We additionally confirmed the functionality of the ACP mutants *in vitro*. We first purified them as 6His-Tev-ACP recombinant proteins. Then, we tested their ability to be acylated with oleate using purified acyl-ACP synthase Aas. The acylation state was then analysed on SDS-PAGE, taking advantage of the aberrant migration of ACP at an apparent molecular weight of 16 kDa on this gel, which is restored to normal (11 kDa) upon acylation. As a control, we used the 6His-Tev-ACP(S36T) protein mutated on the reactive Serine 36 residue, which as a consequence cannot be acylated. We verified that the 6 mutants were acylated by Aas (Figure 2). However, the ACP(F50S) and ACP(I54T) mutants were less effectively acylated than the others (about 85% acylation compared to 25% for the wild type), consistent with the observed growth phenotype of the corresponding mutant strains (see above).

**Figure 3:**
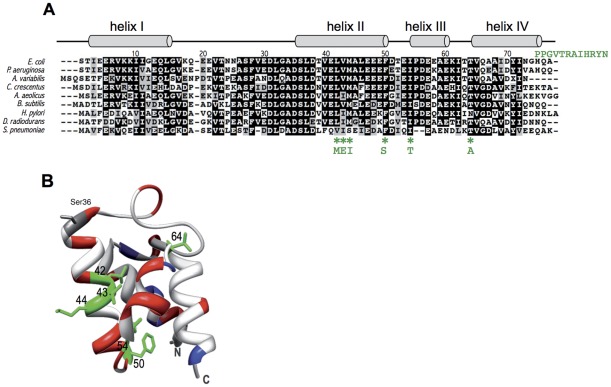
Localization of the mutations in the functional ACP mutants. A. Multiple sequence alignment of selected ACP homologues. The 9 sequences of ACP from the indicated bacteria were aligned with ClustalW [43]. The secondary structure is shown above the alignment. The positions of the mutated residues are indicated with green starts and the substitution is indicated below. The C-terminal extension present in the ACP(T64A, ΔHQA, +12 residues) is shown in green at the right of the alignment. **B.** Ribbon representation of the 3D structure of ACP (PDB 1L0H; [5]) prepared with the UCSF Chimera software [44]. The mutated residues in the different ACP mutants are coloured in green. The essential Ser36 residue, modified by 4′PP and required for ACP function, is highlighted. The acidic and basic residues are coloured in red and blue respectively.

Despite the small size of ACP and its essentiality, we were therefore able to isolate 6 ACP mutants unable to interact with SpoT, but still functional for fatty acid biosynthesis. Three of these mutants had mutations that mapped in consecutive residues of α-helix II: M44I, L42M, and V43E (Figure 3). The mutations in ACP(F50S) and ACP(I54T) mutants located at the bottom of the hydrophobic pocket (Figure 3). Finally, the last mutant carried a T64A substitution together with a frameshift causing a deletion of its 3 last residues replaced by a random C-terminal extension of 12 residues (noted ACP(T64A+Ct), Figure 3).

### Confirmation of the loss of interaction with SpoT using co-purification

The mutants were initially selected for their loss of interaction with SpoT using the bacterial two-hybrid and the plasmid pair pT25-SpoT/pT18-ACP. Yet, we wanted to check the presence or absence of interaction by another technique than the two-hybrid. Previously, the ACP/SpoT interaction was evidenced by affinity co-purification of SpoT with ACP fused to the CBP tag (Calmodulin Binding Peptide) on Calmodulin beads [16]. Yet, we recently showed that the T18 domain of adenylate cyclase used in the two-hybrid has also the property to bind calmodulin beads in presence of calcium [30]. Therefore, we reasoned that we could purify directly the various T18-ACP mutants on Calmodulin beads and test if SpoT was co-purified with them.

**Figure 4:**
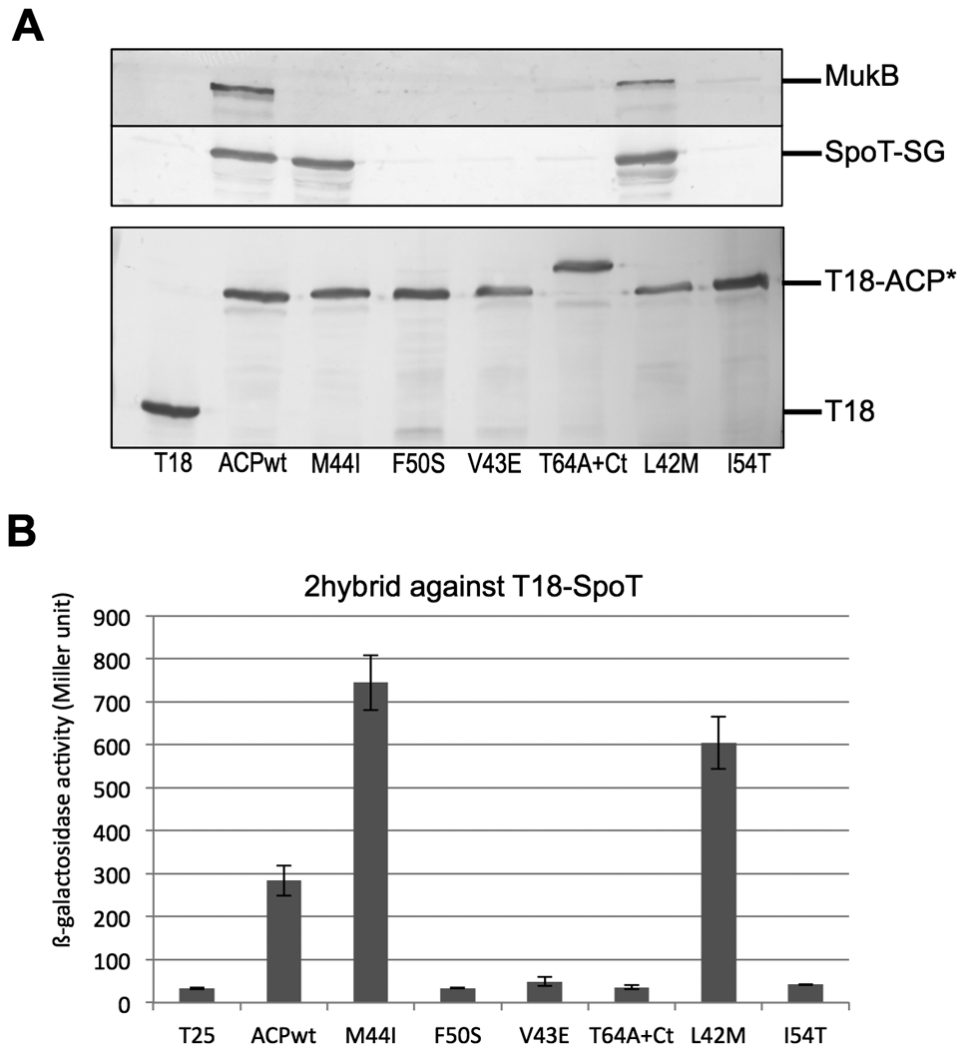
Interactions of the ACP mutants with SpoT and MukB. A. Co-purification on calmodulin beads was performed as described in Experimental procedures on extracts prepared from cultures of W3110SpoT-SG transformed with the indicated pT18-ACP mutant plasmids. Purified samples were analyzed by SDS-PAGE (10% for MukB and SpoT-SG and 12% for T18-ACP) and Western blotting, with anti-T18 to detect the purified ACP mutants, an anti-MukB antibody [45] to detect the wild type endogenous MukB, and finally PAP antibody to detect the endogenous tagged SpoT protein. **B.** BTH101 strain was transformed by pT18-SpoT (pEB596) and the pT25-ACP mutant plasmids. ß-galactosidase assay was performed on 3 independent clones for each pair, as described in the Material and Methods.

We engineered a strain in which SpoT is tagged at its C-terminus by the SG tag (Streptag and IgG binding domains of Protein G separated by the TEV cleavage site) [31]. This permitted us to detect SpoT easily by Western blotting thanks to the Protein G moiety, yet keeping its physiological expression. We first verified that the interaction between T18-ACP and SpoT-SG was detected by affinity purification on calmodulin beads. The W3110/SpoT-SG strain (EB674) was transformed by pT18-ACP and purification on Calmodulin beads was then performed (Material and Methods). SpoT-SG specifically co-purified with T18-ACP as detected by Western blotting (Figure 4A). The W3110/SpoT-SG strain was then transformed by the series of pT18-ACP plasmids corresponding to the ACP mutants that did not interact with SpoT, but that were still functional. Purification on Calmodulin beads was then performed on extracts prepared from each strain.

T18-ACP(F50S), ACP(V43E), ACP(T64A+Ct), and ACP(I54T) did not pull down SpoT-SG (Figure 4A), consistent with the initial two-hybrid screen. Surprisingly, SpoT-SG clearly co-purified with T18-ACP(M44I) and T18-ACP(L42M) mutants (Figure 4A), which were initially selected for the loss of interaction with T25-SpoT in the bacterial two-hybrid test. In a last way of testing the interaction, we transferred the ACP mutations from the pT18 plasmid to the pT25 plasmid and tested the reverse interaction by two-hybrid against T18-SpoT. In this orientation, an interaction was detected only for T25-ACP(M44I) and T25-ACP(L42M) (Figure 4B), therefore giving the same result as the co-purification experiment (Figure 4A). The results of these different tests are summarized in table 2. These results indicated that we had isolated two kinds of mutations provoking distinct effects on the interaction with SpoT: the ones breaking the interaction as evidenced both by two-hybrid and co-purification (F50S, V43E, T64A+Ct, I54T) and the ones breaking only mildly the interaction, only in the case of one combination of the two-hybrid plasmids, and not as assayed by co-purification (L42M and M44I). Because the two-hybrid plasmids have very different copy numbers (much higher for pT18 than pT25), this result suggested that the effect of these two last mutations depended on the stoichiometry of the partners, and especially of SpoT. Indeed, in the co-purification experiment, only SpoT-SG recombinant protein is produced, suggesting that competition with endogenous SpoT might account for the differences in the two-hybrid experiments. To test this hypothesis, we performed the 2-hybrid assay of T18-SpoT against the T25-ACP(L42M) and -ACP(M44I) mutants with or without overexpression of wild type *spoT* in trans. The interaction between SpoT and the two ACP mutants in this combination of plasmids was indeed abolished when *spoT* was overexpressed (Figure S2). We concluded from this experiment that in the T18-ACP/T25-SpoT combination of plasmids used for the initial screen, endogenous wild type SpoT might enter in competition with the low amount of T25-SpoT, thereby increasing the negative effects of the mutations. In the reverse combination (T18-SpoT/T25-ACP), there is much more T18-SpoT produced, which may then not be displaced by the endogenous SpoT.

**Table 2:** Interaction profiles of ACP mutants.

	Two-hybrid T25-				Two-hybrid T18-	Co-purification	
	FabZ	FabA	PlsX	SpoT	SpoT	SpoT-SG	MukB
**ACP**	+	+	+	+	+	+	+
**ACP(M44I)**	−	−	−	−	+	+	−
**ACP(F50S)**	−	−	−	−	−	−	−
**ACP(V43E)**	+	−	−	−	−	−	−
**ACP(T64A+Ct)**	−	−	−	−	−	−	−
**ACP(L42M)**	−	−	−	−	+	+	+
**ACP(I54T)**	−	−	−	−	−	−	−

The two-hybrid interaction profiles have been obtained by assaying the interaction between the T18-ACP mutants and the indicated T25- constructions (first 4 columns), and also by assaying the T25-ACP mutants against T18-SpoT (middle column; Figure 4B). The co-purification results correspond to the experiment presented in figure 4A. The results obtained for the ACP/SpoT interaction in the three different assays (2-hybrid against T18-ACP, 2–hybrid against T25-ACP, and co-purification of SpoT-SG with T18-ACP on Calmodulin beads) are indicated.

### ACP mutations also affect the interaction with other partners of ACP

The mutations that affected SpoT interaction were likely to affect also the interactions with other partners of ACP, even if not abolishing totally its function. In order to test interactions with various known partners of ACP, we used two techniques, affinity co-purification and two-hybrid.

First, we used the exact same samples obtained from the experiment of SpoT-SG pull down by T18-ACP (see above) in order to test the interaction with MukB. This interaction was previously described in the TAP purification of ACP [13]. MukB was clearly identified in the T18-ACP purification (Figure 4A). Only the T18-ACP(L42M) mutant also pulled down MukB protein. In contrast, all the other ACP mutants were impaired in their interaction with MukB (Figure 4A).

In parallel to the co-purification experiment, we tested if it was possible to detect the interaction of ACP with its partner enzymes in fatty acid biosynthesis by the bacterial two-hybrid technique. We cloned nearly all the genes coding for fatty acid synthesis enzymes in the pT25 plasmid and we systematically tested the interaction with ACP. Only T25-FabZ, T25-FabA, and T25-PlsX gave a robust and clear signal of interaction with ACP (Table 2). Therefore, we screened the interaction of the ACP mutants with these 3 proteins by two-hybrid. All the mutants were impaired for the interaction with FabA and PlsX. They were also all affected for the interaction with FabZ, excepted ACP(V43E).

Table 2 summarizes the results obtained by co-purification or by two-hybrid experiments. Different profiles of interactions were observed. ACP(F50S), ACP(T64A+Ct), and ACP(I54T) mutants did not interact with any partner tested. ACP(M44I) and ACP(L42M) were still able to pull down SpoT-SG despite the loss of interaction with T25-SpoT by two-hybrid. ACP(L42M) was the only one to conserve the interaction with MukB and ACP(V43E) was the only one to conserve the interaction with FabZ.

### Phenotypes of the ACP mutants

**Figure 5:**
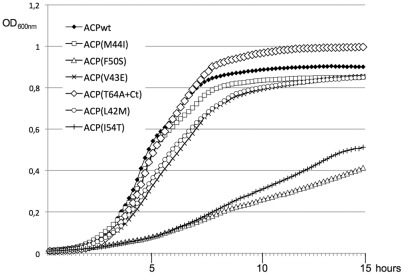
Growth of the ACP mutant strains. Strain MG1665Δ*acpP*::kana^R^ complemented by the pKO3-*acpP* mutant series was grown overnight at 30°C in LB containing Chloramphenicol. The cultures were then diluted 100X in LB containing Chloramphenicol and aliquots of 150 µl were grown in a TECAN microplate reader at 30°C with continuous shaking. Note that the indicated OD_600nm_ is 4 times lower than the usually OD measured with a standard spectrophotometer.

**Figure 6:**
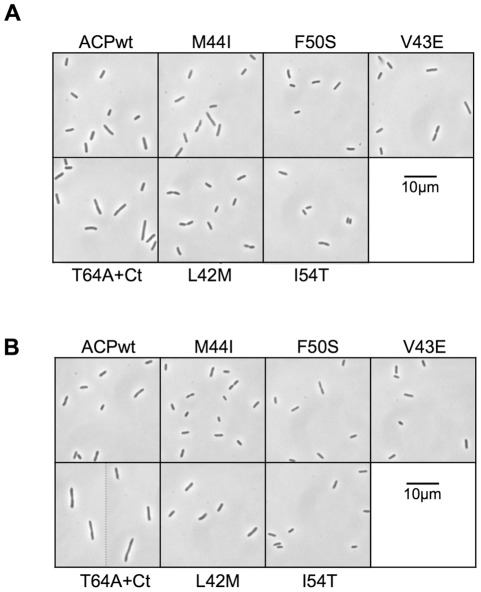
morphology of the ACP mutant strains. Strain MG1665Δ*acpP*::kana^R^ complemented by the pKO3-*acpP* mutant series was grown overnight at 30°C in LB containing Chloramphenicol. The cultures were then diluted 100X in 3 ml of LB containing Chloramphenicol. At OD_600nm_ = 0.5 (**A**) and after overnight growth (**B**), the cultures were observed by phase contrast microscopy with a 100X objective.

It was surprising that mutants unable to interact with enzymes of fatty acid biosynthesis were still able to sustain growth. Therefore, we studied in more detail the growth phenotype of the six MG1655Δ*acpP*/pKO3-ACP mutant strains. As observed on plates, the ACP(F50S) and ACP(I54T) mutants grew very poorly (Figure 5). ACP(V43E) and ACP(L42M) had a lower growth rate than the wild type, while ACP(M44I) and ACP(T64A+Ct) presented the same doubling time in log phase than the wild type. Yet, the ACP(T64A+Ct) mutant presented the interesting behaviour of entering the stationary phase later than the wild type, and reaching a higher final OD_600nm_. This suggested that this mutant was impaired in its response to the entry into stationary phase. Furthermore, when we checked the morphology of the cells, the ACP(T64A+Ct) cells in stationary phase were significantly longer and bigger than the wild type (figure 6), here again showing a failure in the stationary phase program.

The properties of the ACP87(T64+Ct) mutant were the most interesting. Indeed, this ACP mutant protein did not interact with any of the partners of ACP that we had tested (Table 2), yet the corresponding mutant strain grew as well as the wild type. However, it was also the only mutant strain that displayed a morphological phenotype (Figure 6). We further dissected the different mutations present in the ACP87(T64+Ct) mutant in order to understand which mutation caused the loss of interaction with SpoT and the growth phenotype. The ACP(T64A) mutation alone or the deletion of the last 3 residues of ACP alone did not affect the interaction with SpoT, nor the growth of the corresponding mutant strains (Figure S3). In contrast, the random extension of 12 residues caused by the frameshift was sufficient to abolish the interaction with SpoT (Figure S3). However, appearance of the growth and morphology phenotypes required the presence of both the T64A substitution and the C-terminal extension (Figure S3). These results suggest that the C-terminal extension is the main factor affecting the ACP87(T64+Ct) mutant.

One of the goals of this study was to assess the physiological consequences of breaking the ACP/SpoT interaction, especially on the ability of SpoT to increase ppGpp levels in response to fatty acid synthesis inhibition [16], [22]. However, the mutants that we have isolated were not specific for the interaction with SpoT. Nevertheless, we decided to test the ability of the mutant strains MG1655Δ*acpP*/pKO3-ACPwt, -ACP(T64A+Ct), -ACP(M44I), -ACP(V43E), and -ACP(L42M) to synthesize ppGpp in response to fatty acid synthesis inhibition triggered by the cerulenin antibiotic. The four mutants were still able to synthesize ppGpp (Figure S4; and data not shown). This suggested that the loss of SpoT interaction as detected by 2-hybrid and co-purification might not be sufficient to break the SpoT control by ACP, similarly as the loss of several interactions with fatty acid synthesis enzymes was not sufficient to break this synthesis pathway.

## Discussion

There have been extensive studies on the role of specific ACP residues in its folding and its interaction with fatty acid synthesis enzymes (reviewed in [2], [3]). These studies, performed *in vitro*, permitted the prediction of the regions on ACP and on its partner enzymes involved in the interactions. Only recently, the functionality of ACP point mutants has been assayed *in vivo* [24], [27]. The present work brings this approach further, by looking this time at the consequence *in vivo* of ACP mutations on its interactions primarily with SpoT, but also with enzymes of fatty acid synthesis.

In this study, we initially screened for point mutants of ACP unable to interact with SpoT but still functional for fatty acid biosynthesis, as judged by their ability to sustain growth. Three consecutive mutations fulfilling these criteria mapped at the middle of ACP helix II (Figure 3, ACP(M44I), ACP(V43E), ACP(L42M)). This α-helix was already shown to be the site of interaction of ACP with several of its enzyme partners in fatty acid synthesis [2], [8], [9]. Furthermore, the few structures available of ACP in complex with a partner enzyme (FabI, AcpS, YchM) also highlight the importance of helix II in the contact [10]–[12]. However, the mutations not only broke the interaction with SpoT but also abolished the interactions with enzymes involved in fatty acid synthesis such a FabZ, FabA, and PlsX as judged by two-hybrid experiments. These results indicate that ACP helix II is similarly involved in SpoT recognition and in the recognition of fatty acid synthesis enzymes. ACP is a highly acidic protein, and the three central hydrophobic LVM residues of helix II are surrounded by several acidic residues (Figure 3B). This helix is expected to be responsible for the interaction with basic patches at the surface of the partners of ACP [8]. The replacement of the Methionine 44 residue by an Isoleucine residue had an intermediate effect on the interaction with SpoT (table 2) and did not produce any growth phenotype. On the contrary, its replacement by the basic Lysine residues totally abolished ACP function (mutant ACP27, table 1). Similarly, the replacement of Leucine 42 by a Methionine had an intermediate effect on the interaction with SpoT (table 2), and little growth phenotype. These two point mutations correspond to the replacement of large hydrophobic residues by other hydrophobic residues of similar size. This suggests that the overall structure and function of ACP is not affected, but that recognition of SpoT and the fatty acid synthesis enzymes is weakened, consistent with this region being responsible for the recognition of ACP partners. As expected, the mutation of the smaller hydrophobic Valine 43 in a Glutamic acid had more drastic consequences. The interactions were abolished in all the different assays that we have performed (table 2), and a consistent growth phenotype was observed (figure 5). In this case, we can assume that the structure of helix II is more destabilized, hence affecting both the function of ACP and the recognition of its protein partners.

The three residues LVM in α-helix II are mostly conserved in bacteria, except in ACP of *S. pneumoniae* (Figure 3A), whereas SpoT is found only in ß- and γ-proteobacteria. Furthermore, mutations in these three residues similarly affect the interaction with SpoT and with fatty acid synthesis enzymes. This suggests that the specificity of the ACP/SpoT interaction has to be found in SpoT, rather than in ACP. The recognition by the acidic ACP protein of basic patches at the surface of its enzyme partners [8] is consistent with the overall basic nature of SpoT. However, there is no evident enriched cluster of basic residues on this big (702 residues) and complex protein that can be identified without further information. We previously showed that the TGS domain of SpoT is involved in the interaction with ACP [16]. However, this region is no more basic than the rest of the protein. In ß- and γ-proteobacteria, there are two homolog proteins SpoT and RelA that originated from the duplication of an ancestral Rsh protein [32]. We showed that ACP interacted with SpoT, but not with RelA. Yet, when we replaced the TGS domain of SpoT by the TGS domain of RelA, the interaction with ACP was maintained [16]. This showed that the TGS itself, although necessary for the interaction, is not the determinant of the specificity of the interaction with ACP, and that other regions of SpoT must be involved. The recently published phylogenetic study of the RelA/SpoT homolog family will help us to decipher the specificities of SpoT that might account for ACP recognition [32].

The residues mutated in ACP(F50S) and ACP(I54T) mutants localize at the bottom of the hydrophobic pocket, and are important for the folding of ACP and the contact with the fatty acid chain buried in ACP [33]. The corresponding mutant strains are viable, yet their growth is drastically slowed down compared to the wild type strain, and the purified proteins are poorly acylated *in vivo* (Figures 2 and 5). Furthermore, all the interactions with the partners of ACP that we tested were abolished (Table 2). These two residues are absolutely conserved, not only in bacteria (Figure 3A) but also in very distant ACP-like domains that participate in polyketide synthase reactions (See sequence alignments in [24] and in [2]. Consistently, it has been shown that these 2 residues are crucial for maintaining ACP stability [24], [34]. Furthermore, Isoleucine 54 has been implicated in several interactions [9], [10], [35]. In conclusion, the effect of these two mutations on ACP function and on its interaction with SpoT more probably result from a global destabilisation of ACP, and these two residues do not define a specific region for SpoT interaction.

ACP(T64A+Ct) mutant is a special case. In addition to the T64A substitution, it lacks the three last residues of ACP and carries a C-terminal extension, which we showed to be the main determinant of the phenotypes (Figure S3). Despite the total loss of all the interactions tested, it is completely functional. ACP is known to tolerate N-terminal, C-terminal tagging, and even cyclization of its extremities [28]. Yet, despite an initial wild type growth rate during exponential phase, ACP(T64A+Ct) mutant displays a defect in growth regulation as shown by its delayed entry into stationary phase and morphological default. We propose that the C-terminal extension might prevent conformational changes involved in regulation of growth, maybe important for the detection by regulatory proteins such as SpoT.

There is a high degree of sequence conservation between bacterial ACPs involved in fatty acid synthesis (38% identity and 60% similarity for the most distant ACP from *S. pneumoniae* for example), with the DSL signature encompassing the Serine residue modified by the 4′PP group being absolutely conserved (Figure 3A). This reflects the evolutionary constraints imposed on this protein that must interact with so many distinct enzymes. Accordingly, ACPs from various bacterial species can complement an *E. coli* mutant [24], [26]. Yet, we obtained several mutants in conserved residues that were still functional. It has to be noted that our results on residues V43 and I54 are in accordance with a systematic functional analysis of ACP mutants *in vivo* published previously [24]. However, it is very intriguing that without losing the functionality of the ACP mutants, there was a loss of interaction with essential enzymes of fatty acid synthesis. We have assessed the functionality of the mutants for fatty acid synthesis by their ability to sustain growth *in vivo*, but also by their ability to be acylated *in vitro* (Figure 2). This suggests that a limited but sufficient recognition must occur between the ACP mutants and the fatty acid synthesis machinery, but which is not detected by the techniques used to test the interactions.

Clearly, these methods for studying protein-protein interactions showed variation in their sensibility. Despite the first selection for loss of interaction by two-hybrid, the co-purification experiments indicated that the interaction with SpoT is maintained for ACP(M44I) and ACP(L42M) mutants, as it was the case for the reverse two-hybrid experiment (Figure 4). For the co-purification experiments, this discrepancy might be explained by the fact that the interactions with ACP are often maintained by disulphide bridges when the cells are broken, as it was reported before [13], [36], [37]. This stabilization would not happen in the two-hybrid interactions that are detected *in vivo*, explaining the higher sensitivity of this last method to a weakened interaction. In the case of the two combinations of the two-hybrid plasmids, using a competition experiment, we showed that the difference might result from a dependence of the interaction on the stoichiometry between ACP and SpoT and the competition with endogenous SpoT wild type protein (Figure S2). Moreover, the interactions between ACP and its partners are intrinsically weak, because they must be reversible in order to interact and recognize many different enzymes [6]. For fatty acid synthesis, the main determinant of recognition is ultimately the specific substrate of the enzyme bound to the 4′PP group on ACP. The 2-hybrid or co-purification techniques might reflect only the weak and easily broken protein-protein interactions, whereas recognition of the substrate might be sufficient for essential enzymatic steps to occur. The same may apply for the recognition of ACP by SpoT, which is also expected to recognize a specific fatty acid molecule bound to ACP that transduces the information about fatty acid synthesis inhibition to SpoT [16]. This would explain why the ACP mutants are still able to synthesize ppGpp in response to fatty acid synthesis inhibition (Figure S4 and data not shown), as they were also still able to sustain fatty acid synthesis.

It has to be noted that we have used in this study a nearly physiological assay for the functionality of ACP mutants *in vivo* (Figure S5). Indeed, we have used a low copy plasmid ensuring the expression of *acpP* from its natural promoter, without the need for artificial expression. This proved to be important, considering the different results obtained when we tried to complement an *acpP* ^ts^ strain with the high copy number pT18 plasmids (data not shown). The effect of ACP amounts on its function *in vivo* has already been reported [24]. Furthermore, our genetic assay ensures the absence of undesired recombination because the chromosomal copy of *acpP* is completely deleted, and there is no need to grow the cells at high temperature, which permits the study of mutants that might be thermosensitive.

In conclusion, in the light of the initial goals of our study, we were able to determine that helix II of ACP is involved in the recognition of SpoT, by mechanisms similar to the interaction of ACP with enzymes of fatty acid synthesis. Unfortunately, as a consequence, it appeared impossible to specifically break the interaction with SpoT without affecting the recognition of the fatty acid synthesis system. Therefore, it is difficult to assess the physiological consequences of breaking specifically the ACP/SpoT interaction. Yet, we found an ACP mutant that displayed growth regulation phenotypes, confirming our previous hypothesis that lipid metabolism is an important cue in bacterial growth control, as is ppGpp and the stringent response. Further work will be important in order to understand what are the mechanisms of growth control affected in this mutant.

## Materials and Methods

### Media and growth

Cells were grown in Luria-Bertani (LB) medium [38] at 30°C or 37°C as specified in the text. Plasmids were maintained with ampicillin (100 µg/ml), chloramphenicol (30 µg/ml), or kanamycin (50 µg/ml). MacConkey plates contained 50 g/L Difco^TM^ MacConkey agar powder plus ampicillin (100 µg/ml), kanamycin (50 µg/ml) and 1% maltose.

### Plasmids

Plasmids are listed in Table 3. pT18-ACP (pEB379) and pT25-SpoT (pEB595) plasmids used for testing the interaction between ACP and SpoT by bacterial two-hybrid have been described previously [16]. The other two-hybrid plasmids have been constructed by inserting the ORF sequences in pT18 and pT25 plasmids [15], using the oligonucleotides indicated in Table S1. The *acpP* mutant alleles were transferred from the pT18-ACP mutated plasmids to the pT25link plasmid using EcoRI and XhoI restriction sites. Plasmid pCTAP(SG) (pEB1142) was constructed by replacing the TAP tag from pJL148 [39] by the SG tag [31]. pKO3-*acpP* plasmid (pEB1334) was constructed by inserting the *acpP* ORF and the 460 upstream base pairs in the pKO3 low copy plasmid [29] using BamHI and SalI restriction sites. The pKO3-*acpP* mutant plasmids were obtained by site directed mutagenesis on pKO3-*acpP* using the Quickchange kit from Stratagene, for ACP15, ACP68, and ACP132 using the oligonucleotides indicated in table S1. The other mutations were transferred in pKO3-*acpP* by overlapping PCRs: 5′UTR of *acpP* was amplified with Ebm133/136, the *acpP* ORF containing the mutations was amplified with Ebm135/77, and a second PCR performed on these two first PCRs was performed with Ebm133/77 and cloned back in pKO3 (BamHI/SalI). pET-6His-Tev-*acpP* plasmid (pEB1154) and the mutant derivatives were constructed by transferring the *acpP* ORFs in the pET-6His-Tev plasmid (pEB1188) [40] using EcoRI and XhoI restriction sites.

**Table 3:** Plasmids.

Lab code BTH101number	Name	Description	Reference
pEB0354	pT25link	Kana^R^, p15A ori, Plac, *T25*	[15]
pEB0375	pT25-ACP	Kana^R^, p15A ori, Plac, *T25*-*acpP*	[16]
pEB0595	pT25-SpoT	Kana^R^, p15A ori, Plac, *T25*-*spoT*	[16]
pEB1221	pT25-FabZ	Kana^R^, p15A ori, Plac, *T25*-*fabZ*	This work
pEB0721	pT25-FabA	Kana^R^, p15A ori, Plac, T25-fabA	This work
pEB0861	pT25-PlsX	Kana^R^, p15A ori, Plac, T25-plsX	This work
pEB0355	pT18link	Amp^R^, ColE1 ori, Plac, T18	[15]
pEB0379	pT18-ACP	Amp^R^, ColE1 ori, Plac, T18-acpP	[15]
pEB0596	pT18-SpoT	Amp^R^, ColE1 ori, Plac, T18-spoT	[16]
pEB0999	pACYC-P_BAD_-*spoT*	Cam^R^, p15A ori	This work
pEB1188	pET-6His-Tev	Amp^R^, T7 promoter, 6His-Tev N-terminal tag	[40]
pEB1154	pET-6His-Tev-ACP	acpP PCR Ebm76/77 in pEB1188(EcoRI/XhoI)	This work
pEB0817	pET28-Aas-6his		[42]
pEB1123	pCEMM-CTAP(SG)	AmpR, ColE1 ori, CTAP(SG) tag	[31]
pEB0794	pJL148	SPA-FRT-kanaR-FRT	[39]
pEB1142	pCTAP(SG)	TAP(SG)-kanaR-FRT	This work
pEB0270	pKD13	Amp^R^, FRT-flanked Kana^R^	[41]
pEB0267	pKD46	R101 ori (ts), Amp^R^, lambda Red genes	[41]
pEB0232	pKO3	*repA*(ts) ori, Cam^R^, M13 ori, *sacB*	[29]
pEB1334	pKO3-*acpP*	*acpP* PCR Ebm133/77(BamHI/XhoI) in pKO3(BamHI/SalI)	This work
pEB1369	pKO3-acpP(S36T)	S36T mutagenesis on pEB1334	This work

ts: thermosensitive replication; Amp^R^: carrying resistance to Ampicillin gene, Kana^R^; carrying resistance to Kanamycin gene; Cam^R^: carrying resistance to Chloramphenicol. ori: origin of replication; FRT: recombination site for Flipase. The plasmids containing the mutations in *acpP* are not listed. They correspond to mutagenesis of pEB379, pEB375, pEB1154, and pEB1334 plasmids.

### Strains

All strains used are derivatives of *E. coli* K12 and are listed in table 4. The *cya* strain BTH101 [25] was used for the two-hybrid assay. The MG1655*acp* ^ts^ strain (EB341) was used to test the functionality of the T18-ACP recombinant proteins [26]. Strain MC4100 was used for testing T18 tagged protein expression. Finally, strain C600 was used for plasmid constructions.

**Table 4:** *E. coli* K12 strains.

Lab code BTH101number	Name	Description	Reference
–	MG1655	F^-^ λ^-^ *ilvG- rfb-50 rph-1*	[46]
–	C600	F^-^ *tonA21 thi*-1 *thr*-1 *leuB6 lacY1 glnV44 rfbC1 fhuA1* λ^-^	[46]
–	W3110	F^-^ λ^-^ INV(*rrnD*, *rrnE*) *rph*-1	[46]
EB003	BTH101	F- *cya*-99 *araD139 galE15 galK16 rpsL1*(Str^R^) *hsdR2 mcrA1 mcrB1 relA1*	[25]
EB341	MG1655 *acpP^ts^*	*acpP^ts^* Δ*fabF*::cam^R^	[26]
EB674	W3110/SpoT-SG	*spoT*-SG-FRT-kana^R^-FRT	This work
**EB689**	**MG1655Δ** ***acpP*** ** pKO3-** ***acpP***	**MG1655Δ** ***acpP*** **::kana^R^ + pEB1334**	**This work**
EB724	MG1655Δ*acpP* pKO3-*acpP(M44I)*	MG1655Δ*acpP*::kana^R^ + pEB1365	This work
EB725	MG1655Δ*acpP* pKO3-*acpP(F50S)*	MG1655Δ*acpP*::kana^R^ + pEB1370	This work
EB726	MG1655Δ*acpP* pKO3-*acpP(V43E)*	MG1655Δ*acpP*::kana^R^ + pEB1360	This work
EB727	MG1655Δ*acpP* pKO3-*acpP(T64A+Ct)*	MG1655Δ*acpP*::kana^R^ + pEB1377	This work
EB728	MG1655Δ*acpP* pKO3-*acpP(L42M)*	MG1655Δ*acpP*::kana^R^ + pEB1362	This work
EB729	MG1655Δ*acpP* pKO3-*acpP(I54T)*	MG1655Δ*acpP*::kana^R^ + pEB1391	This work

Ts: thermosensitive. Kana^R^; carrying resistance to Kanamycin gene; Cam^R^: carrying resistance to Chloramphenicol. FRT: recombination site for Flipase.

W3110/SpoT-SG strain was constructed by recombining the SG tag sequence downstream *spoT* gene using pCTAP(SG) (pEB1142) plasmid and the Datsenko and Wanner method [41]. MG1655Δ*acpP::kana^R^*/pKO3-*acpP* strain was obtained by transforming MG1655 by pKO3-*acpP* and pKD46 and then deleting the chromosomal copy of *acpP* by recombination. The various *acpP* mutant strains were then constructed by introducing the Δ*acpP*::*kana* ^R^ mutation in MG1655 strain transformed by the various pKO3-*acpP* mutants using generalized P1 phage transduction. The deletion of the *acpP* allele in the resulting MG1655/pKO3-*acpP* mutant strains and the absence of duplication events were verified by PCR (Figure S6). We also checked by PCR and sequencing that the plasmids themselves were unchanged. The amount of ACP protein produced in this strain was compared with the amount produced from the chromosomal copy of *acpP* using ACP-TAP tagged clone and strain (see Figure S3).

### Bacterial two-hybrid

After co-transformation of BTH101 strain [25] with the two plasmids expressing the T18- and T25- fusions, LB plates containing ampicillin and kanamycin were incubated at 30°C for 2 days. 3 ml of LB medium supplemented with ampicillin, kanamycin and 0.5 mM IPTG were inoculated and grown at 30°C overnight. ß-Galactosidase activity was determined as described [38]. The values presented are the mean of 3 or 4 independent assays.

### *acpP* mutagenesis

We performed random mutagenesis on the *acpP* gene ORF using the GeneMorph II Random Mutagenesis Kit from Stratagene with oligonucleotides Ebm76/77. The PCR was then cloned in the pT18link (pEB355) plasmid [15] using EcoRI and XhoI restriction sites. Strain BTH101 already transformed by pT25-SpoT was transformed by the resulting library of pT18-ACP mutants, and plated on LB plates containing Ampicillin and Kanamycin. After 2 days at 30°C, colonies were replicated on MacConkey petri dishes and again incubated 2 days at 30°C. Minipreps of DNA were prepared from the white colonies and used to transform an MC4100 strain in order to reisolate the pT18-ACP mutant alone. A new two-hybrid assay was performed between the isolated T18-ACP mutant and T25-SpoT in BTH101 strain in order to verify the loss of interaction. Then the expression of the recombinant T18-ACP mutant proteins was verified by Western blot using the 3D1 antibody directed against the T18 domain. Clones giving full-length T18-ACP proteins were sequenced and kept for further analysis.

### In vitro acylation assay of purified ACP mutants

6His-Aas was purified as described before using the pET28-Aas-6his plasmid [42]. All the ACP mutants were purified as 6His-Tev-ACP recombinant proteins, as described before [40]. The acylation reaction (100 mM Tris pH 8, 5 mM DTT, 10 mM ATP, 10 mM MgSO4, 100 µg/ml oleate, 2 µg ACP proteins, and 0.5 µg 6His-Aas in a final volume of 20 µl) was incubated 1 hour at 37°C. The ACP species were then analyzed on a 15% SDS-PAGE stained with Coomassie Blue. In this gel, acylated ACP proteins migrate faster than the holo-ACP.

### Co-purification on Calmodulin beads

From a 200 ml culture grown at 37°C in LB and induced during 120 min with 0.5 mM IPTG to a final OD_600_ of approximately 2, an extract was prepared by sonication in 7 ml Calmodulin Binding Buffer (10 mM Tris-HCl pH 8.0, 150 mM NaCl, 0,1% NP40, 1 mM Mg-acetate, 1 mM imidazole, 2 mM CaCl_2_,) with 0.75 µM PMSF. After centrifuging 30 min at 27000 g, glycerol was added to 15% final concentration and the extract was frozen in liquid nitrogen. For each co-purification assay, 3 ml of extract were incubated on 40 µl of Calmodulin beads previously washed in Calmodulin Binding Buffer. After 60 min of incubation at 4°C, the beads are washed fives times in 1 ml of Calmodulin binding buffer, resuspended in 40 µl of Laemmli loading buffer, and heated 5 min at 96°C.

### SDS-PAGE and western blot

SDS-PAGE, electrotransfer onto nitrocellulose membranes, and Western-blot analyses were performed as previously described [13]. SG tag was detected with the PAP antibody from Sigma. Monoclonal anti-T18 3D1 was purchased from Santa Cruz.

## Supporting Information

Figure S1 **Production of the T18-ACP mutant proteins.** Strain MC4100 was transformed with the indicated pT18-ACP mutants. After induction for 3 h with 0.5 mM IPTG in LB medium at 37°C, the recombinant proteins were detected by Western blotting on a 10% SDS-PAGE using anti-T18 monoclonal antibody (3D1-Santa Cruz).
(PDF)

Figure S2 **Competition assay of the interaction between T18-SpoT and the T25-ACP mutants**: Strain BTH101 was transformed with pT18-SpoT (pEB596) and the indicated pT25-ACP plasmid (pEB375 and its mutant derivatives), together with the third pACYC-PBAD-SpoT plasmid (pEB999, +) or the control pACYC184 (−). Cultures were grown overnight at 30°C in LB supplemented with ampicillin, kanamycin, chloramphenicol, 0.5 mM IPTG to induce the expression from the 2-hybrid plasmids, and 0.5% arabinose to induce expression of *spoT* in trans from the pACYC-PBAD-SpoT plasmid. ß-Galactosidase activity was determined as described [38]. The values presented are the mean of 3 independent assays.
(PDF)

Figure S3 **Dissection of the mutations in the ACP(T64A+Ct) mutant. A. Interaction with SpoT.** The T64A substitution, the C-terminal extension, and the deletion of the last 3 residues of ACP were separated in three new T18-ACP constructions and assayed for interaction with T25-SpoT. **B. Growth of the mutant strains.** The same mutations were introduced in pKO3-*acpP* plasmid and the corresponding mutant strains were constructed by P1 transduction of the Δ*acpP*::kana^R^ allele. The growth of the mutant strains was followed at 30°C in a TECAN microplate reader.
(PDF)

Figure S4 **(p)ppGpp synthesis assay in response to fatty acid synthesis inhibition by cerulenin.** (p)ppGpp was measured accordingly to [16]. In brief, cultures in low-phosphate medium of the EB689 and EB727 strains were continuously labeled for at least two generations with 100 µCi of [^32^P]orthophosphate per ml starting at DO600 = 0.05. Cerulenin was then added at a final concentration of 200 µg.ml^−1^, and 20 µl of samples were taken at time 0, 3, 8, 15, 30, and 45 minutes. Samples were immediately mixed with 20 µl of 16 M formic acid on ice. 5 µl of the acid formic extracts were chromatographed in one dimension on 20×10 cm polyethyleneimine cellulose TLC plates (JT Baker). TLC plates were developed using a FLA5100 Fuji phosphorimager.
(PDF)

Figure S5 **Comparison of the amount of ACP-TAP protein in strains MG1655/ACP-TAP and MG1655Δ** ***acpP*** **::kana^R^ complemented by pKO3-** ***acpP*** **-TAP.** **A,** **C**: MG1655/ACP-TAP (EB657); **B,** **D**: MG1655Δ*acpP*::kana^R^/pKO3-*acpP*-TAP (pEB767). For each strain, cultures in 20 ml LB containing ampicillin (100 μg/ml) were incubated at 30°C with agitation for 5 h (DO_600_ A = 1.6 and DO_600_ B = 1) and 24 h (DO_600_ C = 6 and DO_600_ D = 6.7). Cells were pelleted by centrifugation and total cell extracts were analyzed on a 10% SDS-PAGE followed by Western-blot with PAP antibody (Sigma).
(PDF)

Figure S6 **Control of the Δ** ***acpP*** **::kana^R^ deletion in MG1655/pKO3-** ***acpP*** **mutant strains.** The transduction of the Δ*acpP*::kana^R^ allele in the MG1655/pKO3-*acpP* mutant strains was verified by PCR on colonies using oligonucleotides ebm220/ebm710. Amplification of a fragment of 1510 bases pairs indicates that strains have acquired the Δ*acpP*::kana^R^ allele; amplification of 411 pb fragment indicates strains carrying the wild type *acpP* locus. Ebm220: 5′- ATTTTATACACTACGAAAACCATCGCG -3′Ebm710: 5′- ACGGGATCCTCCGGTCACAACTACACGACG -3′.
(PDF)

Table S1 **Oligonucleotides used in this study.**
(PDF)
