# Supplementary figures and images for: Disrupting the Acyl Carrier Protein/SpoT Interaction In Vivo: Identification of ACP Residues Involved in the Interaction and Consequence on Growth
Source: PLoS One. 2012 Apr 30;7(4):e36111. doi: 10.1371/journal.pone.0036111 (PMC3340395; doi:10.1371/journal.pone.0036111)

Figure S1

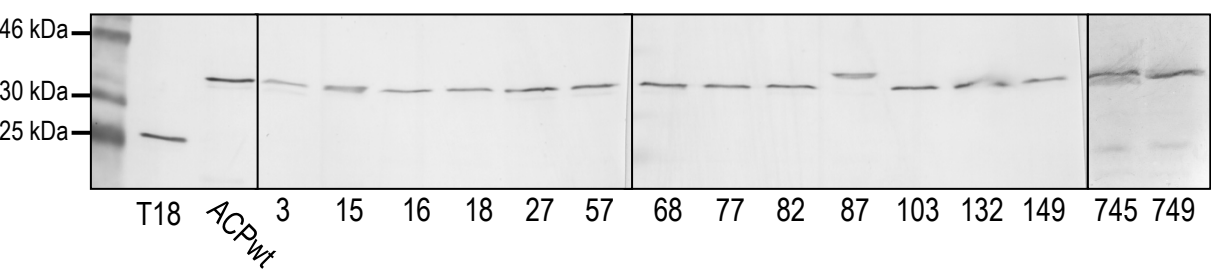

Supplement: Figure S1 — Production of the T18-ACP mutant proteins. Strain MC4100 was transformed with the indicated pT18-ACP mutants. After induction for 3 h with 0.5 mM IPTG in LB medium at 37°C, the recombinant proteins were detected by Western blotting on a 10% SDS-PAGE using anti-T18 monoclonal antibody (3D1-Santa Cruz). (PDF) [file pone.0036111.s001.pdf]

Figure S2

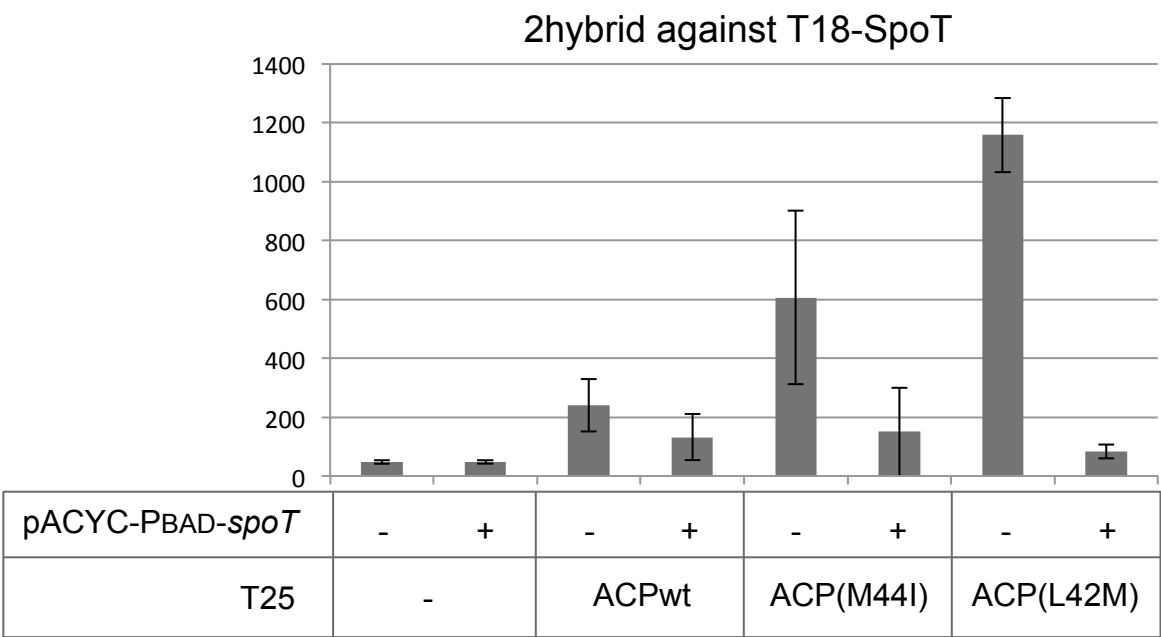

Supplement: Figure S2 — Competition assay of the interaction between T18-SpoT and the T25-ACP mutants: Strain BTH101 was transformed with pT18-SpoT (pEB596) and the indicated pT25-ACP plasmid (pEB375 and its mutant derivatives), together with the third pACYC-PBAD-SpoT plasmid (pEB999, +) or the control pACYC184 (−). Cultures were grown overnight at 30°C in LB supplemented with ampicillin, kanamycin, chloramphenicol, 0.5 mM IPTG to induce the expression from the 2-hybrid plasmids, and 0.5% arabinose to induce expression of spoT in trans from the pACYC-PBAD-SpoT plasmid. ß-Galactosidase activity was determined as described [38]. The values presented are the mean of 3 independent assays. (PDF) [file pone.0036111.s002.pdf]

Figure S3

A

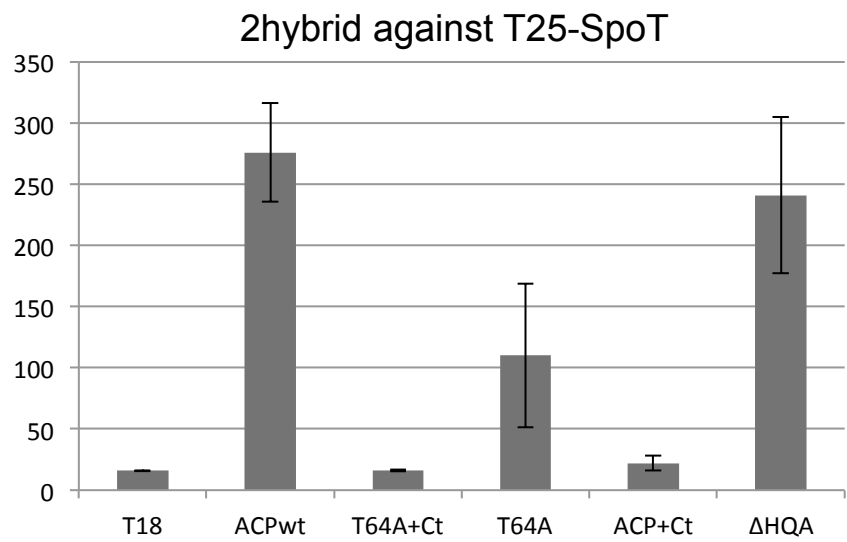

B

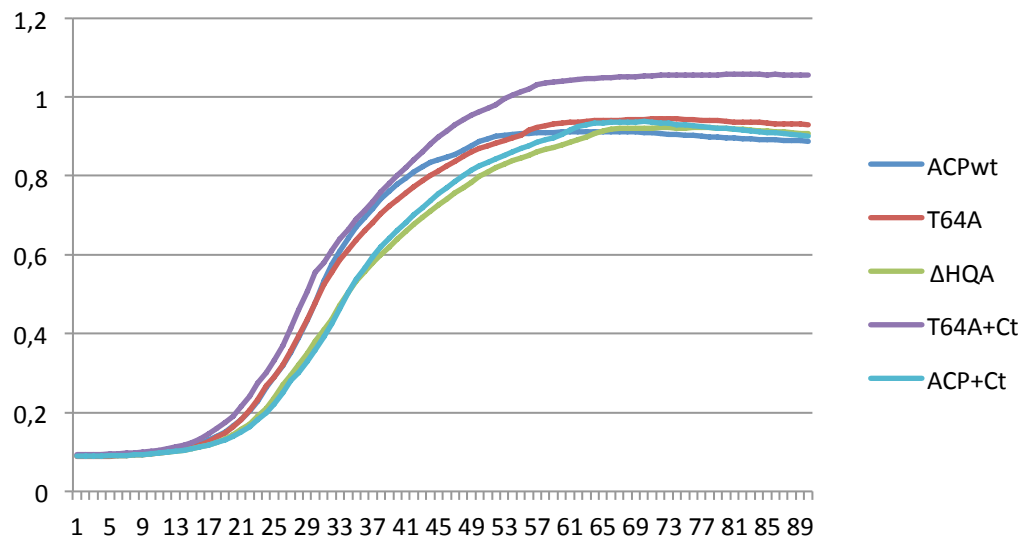

Supplement: Figure S3 — Dissection of the mutations in the ACP(T64A+Ct) mutant. A. Interaction with SpoT. The T64A substitution, the C-terminal extension, and the deletion of the last 3 residues of ACP were separated in three new T18-ACP constructions and assayed for interaction with T25-SpoT. B. Growth of the mutant strains. The same mutations were introduced in pKO3-acpP plasmid and the corresponding mutant strains were constructed by P1 transduction of the ΔacpP::kanaR allele. The growth of the mutant strains was followed at 30°C in a TECAN microplate reader. (PDF) [file pone.0036111.s003.pdf]

**Figure S4**

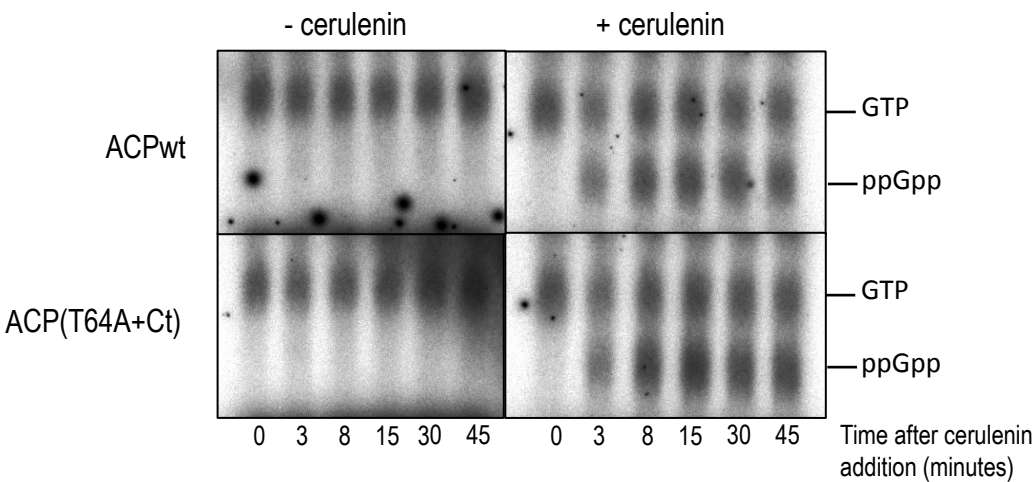

Supplement: Figure S4 — (p)ppGpp synthesis assay in response to fatty acid synthesis inhibition by cerulenin. (p)ppGpp was measured accordingly to [16]. In brief, cultures in low-phosphate medium of the EB689 and EB727 strains were continuously labeled for at least two generations with 100 µCi of [32P]orthophosphate per ml starting at DO600 = 0.05. Cerulenin was then added at a final concentration of 200 µg.ml−1, and 20 µl of samples were taken at time 0, 3, 8, 15, 30, and 45 minutes. Samples were immediately mixed with 20 µl of 16 M formic acid on ice. 5 µl of the acid formic extracts were chromatographed in one dimension on 20×10 cm polyethyleneimine cellulose TLC plates (JT Baker). TLC plates were developed using a FLA5100 Fuji phosphorimager. (PDF) [file pone.0036111.s004.pdf]

**Figure S5**

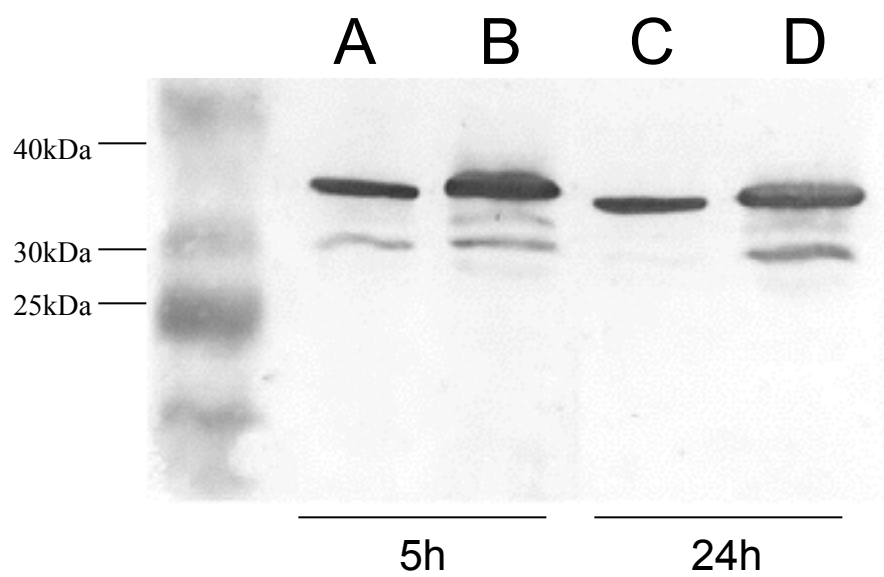

Supplement: Figure S5 — Comparison of the amount of ACP-TAP protein in strains MG1655/ACP-TAP and MG1655Δ acpP ::kanaR complemented by pKO3- acpP -TAP. A, C: MG1655/ACP-TAP (EB657); B, D: MG1655ΔacpP::kanaR/pKO3-acpP-TAP (pEB767). For each strain, cultures in 20 ml LB containing ampicillin (100 μg/ml) were incubated at 30°C with agitation for 5 h (DO600 A = 1.6 and DO600 B = 1) and 24 h (DO600 C = 6 and DO600 D = 6.7). Cells were pelleted by centrifugation and total cell extracts were analyzed on a 10% SDS-PAGE followed by Western-blot with PAP antibody (Sigma). (PDF) [file pone.0036111.s005.pdf]

Figure S6

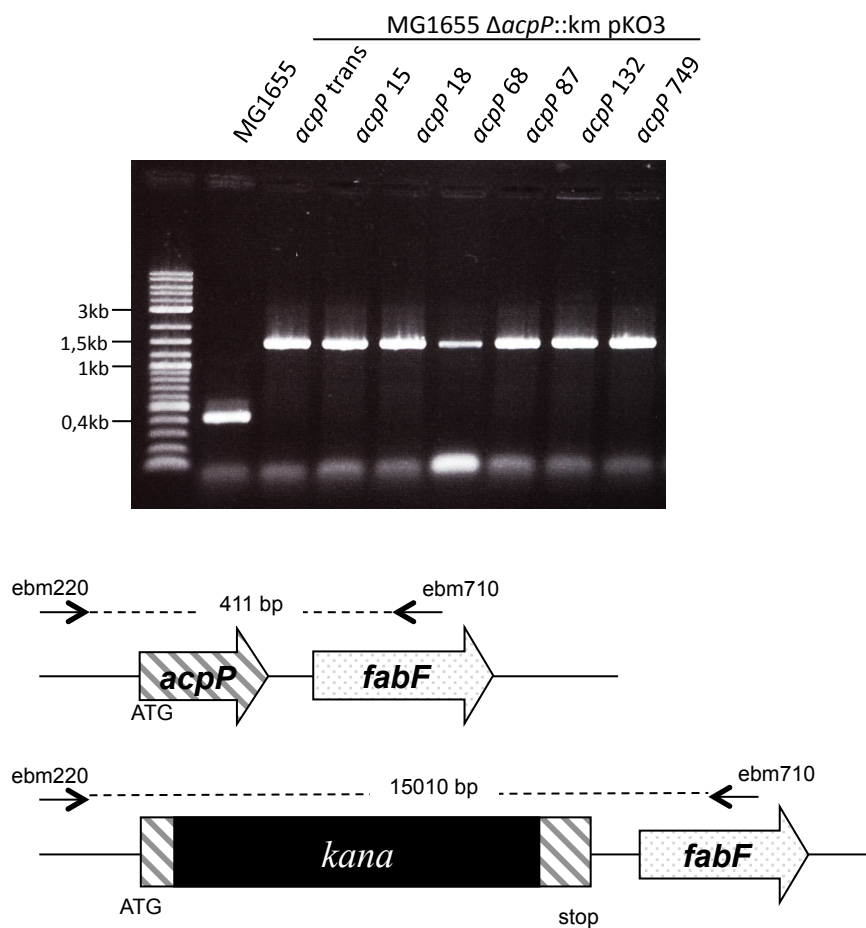

Supplement: Figure S6 — Control of the Δ acpP ::kanaR deletion in MG1655/pKO3- acpP mutant strains. The transduction of the ΔacpP::kanaR allele in the MG1655/pKO3-acpP mutant strains was verified by PCR on colonies using oligonucleotides ebm220/ebm710. Amplification of a fragment of 1510 bases pairs indicates that strains have acquired the ΔacpP::kanaR allele; amplification of 411 pb fragment indicates strains carrying the wild type acpP locus. Ebm220: 5′- ATTTTATACACTACGAAAACCATCGCG -3′Ebm710: 5′- ACGGGATCCTCCGGTCACAACTACACGACG -3′. (PDF) [file pone.0036111.s006.pdf]
