# Supplementary material for: Disrupting the Acyl Carrier Protein/SpoT Interaction In Vivo: Identification of ACP Residues Involved in the Interaction and Consequence on Growth
Source: PLoS One. 2012 Apr 30;7(4):e36111. doi: 10.1371/journal.pone.0036111 (PMC3340395; doi:10.1371/journal.pone.0036111)
Supplement: Table S1 — Oligonucleotides used in this study. (PDF) [file pone.0036111.s007.pdf]

Lab	5' -> 3' sequence	use
Ebm76	CACC**GAATTC**ATGAGCACTATCGAA	Random *acpP* mutagenesis
Ebm77	ACCG**CTCGAG**TTACGCCTGGTGGCC	Random *acpP* mutagenesis
Ebm133	GACCGGATCCAAATCACTGGCGCGCGAAG	pKO3-acpP
Ebm135	ATGAGCACTATCGAAGAACGC	pKO3-*acpP* mutants
Ebm136	GCGTTCTTCGATAGTGCTCAT	pKO3-*acpP* mutants
Ebm730	AAAACCATCGCGAAAGCGAGTTTTGATAGGAAATTTAAGAGT ATG**ATTCCGGGGATCCGTCGACC**	Δ*acpP*::kana
Ebm731	GAACGACCGCCTGGAGATGTTCACTTACGCCTGGTGGCCGTT GAT**TGTAGGCTGGAGCTGCTTCG**	Δ*acpP*::kana
Ebm502	ACT**GCTAGC**ATGGACGAGAAGACCACCGG	Plasmid TAP(SG)
Ebm503	CAC**GATAT**CTATTCAGTGACAGTGAAAGTCTTTG	Plasmid TAP(SG)
Ebm518	GTGATGCCAGACGTGATTAAAGTCACCCGAAACCGAAATGCT AGCATGGACGAGAAGACC	*spoT*-SG FW
Ebm341	GAGCATTTCGCAGATGCGTGCATAACGTGTTGGGTTCATAAA ACACATATGAATATCCTCCTTAG	*spoT*-SG RV
Ebm666	CACCGTTGAGCTGGTAAT**AGCGCT**GGAAGAAGAGTTTGATAC	Met44Ile
Ebm667	GTATCAAACTCTTCTTCC**AGCGCT**ATTACCAGCTCAACGGTG	Met44Ile
Ebm676	CCGTTGAGCTGGAAATGGCTCTAGAAG	Val43Glu
Ebm677	CTTCTAGAGCCATTTCCAGCTCAACGG	Val43Glu
Ebm699	CCGTTGAGATGGTAATGGCTCTAGAAGAAG	Leu42Met
Ebm700	CTTCTTCTAGAGCCATTACCATCTCAACGG	Leu42Met
Ebm739	GCTTCTTTCGTTGAA**T**ACCT**A**GGCGCGGATTCTCTT	Asp31Tyr
Ebm740	AAGAGAATCCGCGCC**T**AGGT**A**TTCAACGAAAGAAGC	Asp31Tyr
Ebm741	TTGAGCTGGTAATGG**T**TCT**A**GAAGAAGAGTTTGAT	Ala45Val
Ebm742	ATCAAACTCTTCTTC**T**AGA**A**CCATTACCAGCTCAA	Ala45Val
Ebm745	CCACCGTTCAGGCTG**A**CAT**C**GATTACATCAACGGC	Ala68Asp
Ebm746	GCCGTTGATGTAATC**G**ATG**T**CAGCCTGAACGGTGG	Ala68Asp
